# Psychosocial Support in Liver Transplantation: A Dyadic Study With Patients and Their Family Caregivers

**DOI:** 10.3389/fpsyg.2019.02304

**Published:** 2019-10-10

**Authors:** Sabrina Cipolletta, Lorenza Entilli, Massimo Nucci, Alessandra Feltrin, Giacomo Germani, Umberto Cillo, Biancarosa Volpe

**Affiliations:** ^1^Department of General Psychology, University of Padua, Padua, Italy; ^2^Psychology Service, Padua University Hospital, Padua, Italy; ^3^Multivisceral Transplant Unit, Department of Surgery, Oncology and Gastroenterology, Padua University Hospital, Padua, Italy; ^4^Hepatobiliary Surgery and Liver Transplantation Unit, Department of General Surgery and Organ Transplantation, Padua University Hospital, Padua, Italy

**Keywords:** caregiver, dependency, dyad, liver transplantation, social support

## Abstract

**Background and aims**: Liver transplantation provides an opportunity of survival for patients with liver failure; however, this procedure is known to be psychologically and physically fatiguing for patients and their informal caregivers. The aim of this study was to investigate how perceived social support and the distribution of dependency were associated with the psychological wellbeing of patients waiting for liver transplantation and their caregivers, as a dyad.

**Methods**: The present was a cross-sectional study. Ninety-five participants were recruited at a hospital in Northern Italy, during the psychological evaluation for inclusion in the transplantation list: 51 patients (19 with alcohol-related illness) and 44 family caregivers. Both patients and caregivers filled in a Symptom Checklist and Kelly’s Dependency Grids. Patients also compiled the Medical Outcome Study Social-Support Survey, and caregivers compiled the Family Strain Questionnaire Short-Form.

**Results**: Caregivers reported important levels of strain and strongly related to a worsening of their own and patients’ symptoms. Patients with alcohol-related pathologies had a narrower social network, which corresponded to an increase in family strain. On the sample as a whole, regression analyses showed that perceived social support and dependency measures did not predict patients’ and caregivers’ symptoms. Nevertheless, cluster analysis identified a group of caregivers who distributed their dependency more and experienced lower levels of depression, anxiety, and strain.

**Conclusions**: These results suggest the usefulness of a dyadic approach in the research, prevention, and care of liver diseases. A deeper comprehension of the functioning of dyads will help practitioners in the identification of situations at risk.

## Introduction

Liver transplantation is a well-established treatment that provides an opportunity of survival in the occurrence of liver failure (Duffy et al., 2010; Dew et al., 2015). Liver failure may be due to a metabolic cause, autoimmune response causes, viral causes (such as Hepatitis B and C or cirrhosis of unknown origin), and alcohol or drug abuse (Sarin et al., 2009; Charlton et al., 2011).

The quality of life of patients who suffer from a liver disease is often impaired by the progression of the disease and burdened by the presence of disease-related complications, restrictions on social life, and strict compliance to a medication regimen (De Bona et al., 2000). Physical symptoms involve marked worsening of overall health (Lai et al., 2015), nausea, sleepiness, mental disorientation, and confusion, depending on the severity of the illness (Onyekwere et al., 2011) and fatigue (Derck et al., 2015), all of which usually recede after the transplant (Duffy et al., 2010). However, effects on cognitive functioning and other psychological outcomes may be more long-lasting (Malik et al., 2014; Nguyen et al., 2015), including the insurgence of high levels of anxiety and depression (Dew et al., 2015).

From the diagnosis of organ failure to the operation, transplant recipients go through an articulated process, which is known to be psychologically and physically fatiguing both for them (Kimbell et al., 2015) and their informal caregivers (Cohen et al., 2007; Rodrigue et al., 2010, 2011; Goetzinger et al., 2012). Although substance-abuse-related liver disease and associated risky behavior are already linked to long-standing family conflicts and subsequent deterioration in relationships (Mangueira and Lopes, 2016; Hansen et al., 2017; Le et al., 2019), other types of liver disease may also change the patient’s mood and behavior, thus making the patient becomes less lucid and, occasionally, more aggressive (Jim et al., 2014). Lower income level, unemployment, and living with spouse are positively associated with having depression in patients suffering from chronic hepatitis B (Vu et al., 2019a).

Caregivers of patients awaiting solid organ transplantation end up reporting somatic complaints and greater affective distress, including exhaustion, anger, depression, and feelings of anxiety (Goetzinger et al., 2012). Furthermore, informal caregivers consistently experience more distress when their own social support networks are limited (Armoogum et al., 2013).

Social support is a multidimensional construct referring to the availability of social resources in a specific context. Different close relationships may provide different forms of support, such as emotional support, instrumental support, informational support, and positive support (Giangrasso and Casale, 2014). Boscarino et al. (2015) highlighted how experiencing higher stressful life events and lower social support represented a risk factor for poor health among patients with chronic hepatitis C infection. Also, perceived low social support by the patient is an important risk factor for their commitment to follow a treatment regimen (Rodrigue et al., 2013), and in general, psychosocial vulnerability is a valid predictor of the success of a transplant operation (Goetzmann et al., 2007; Goetzinger et al., 2012). On the other hand, social support and good psychological functioning of caregivers of liver transplantation patients represent a valid protective factor (Goetzinger et al., 2012; Goetzmann et al., 2012; Malik et al., 2014). As a matter of fact, social support emerged as one of the most influential factors, among transplant providers, in determining patients’ suitability for transplantation (Ladin et al., 2018). In light of that, and because of the high involvement in the patients’ care and its potential impact on patients’ long-term outcomes (Nguyen et al., 2015), the caregivers’ health should also be taken into consideration and protected.

In the existing literature regarding organ transplantation, few quantitative studies (Goetzmann et al., 2012; Malik et al., 2014) confront the experiences of the two members of the patient-caregiver dyad by taking into consideration how they both play an important role in improving and maintaining health. Malik et al. (2014) only compared patients’ and caregivers’ scores, without exploring the possible relationships of the anxiety and depression rates with other aspects of the relationship within the dyad. Goetzmann et al. (2012) conducted a more in-depth analysis but included patients and spouses that had already undergone organ transplantations without discriminating among different organs. Little is still known about how patients and caregivers influence each other in the organ transplantation waiting experience (Hansen et al., 2017).

Medical outcome variables such as survival rates and specific morbidity rates are widely accepted parameters in clinical studies. However, “the efficacy of any operation must be evaluated not only by perioperative complications and long-term survival rates, but also by the effect on the physical, psychological, emotional, and social wellbeing of patients” (Duffy et al., 2010, p. 652).

Kelly’s personal construct psychology might prove useful for this aim (Kelly, 1955; Hermann et al., 2017). When personal constructs are implied in the maintenance of one’s own basic needs, they are referred to as core constructs. Some of these – the dependency constructs – ensure our survival by allowing the satisfaction of our needs within our close relationships, as the satisfaction of the need for food, protection, and care testifies. Contrary to conventional wisdom, the ability to ask for help and rely on others is not a characteristic typical only of infants; as a matter of fact, everyone depends on someone else for something. The distribution of dependency allows people to differentiate among their resources, so that some resources meet some needs, while others satisfy different needs (Walker, 2003). This concept is central to the ways we live and cope with illness (Cipolletta et al., 2012, 2017) and ask and receive care (Cipolletta et al., 2013).

The aim of this study was to investigate how perceived social support and the distribution of dependency were associated with the psychological wellbeing of patients waiting for liver transplantation and their caregivers. Specifically, our hypotheses were that:

1. an increase in patients’ psychological symptoms will correlate with an increase in caregivers’ psychological symptoms and strain;
2. an increase in social support will correlate with a decrease in patients’ and caregivers’ psychological symptoms;
3. the breadth of patients’ and caregivers’ networks and a higher distribution of dependency will correlate with a decrease in their psychological symptoms and in the family strain;
4. the way patients and caregivers rely on themselves or each other will relate to a decreased symptomatology or strain;
5. there will be an association between some measures of social support and the distribution of dependency;
6. there will be an association between some measures of dependency and depression, anxiety, and strain; and
7. the subgroup of dyads composed of patients with an alcohol-related disease will emerge as more challenged than dyads facing a liver disease with metabolic origin.

## Materials and Methods

### Study Setting and Sample

A convenience sample of 95 participants was recruited at the Liver Transplantation Center of Padova Hospital, during the psychological evaluation for inclusion in the transplantation list. Of these, 51 were patients (10 women and 41 men), and 44 were their respective caregivers (36 women and 8 men). Seven patients (two women and five men) arrived at the hospital without a relative or an informal caregiver, in some cases declaring that they felt healthy enough to travel alone, in others that they did not have one. The decision to include them in the total number of participants was made because their experience was considered informative, although, of course, their data were excluded when dyadic analysis between the couples was conducted.

The inclusion criteria were willing and able to provide informed consent, being >18 years old, having enough knowledge of the Italian language, and, for the patients, being eligible for a liver transplantation according to the EASL (European Association for the Study of the Liver) guidelines, and being sufficiently lucid to complete the tests (that is, not suffering from encephalopathy at the moment of the testing) according to the EASL (European Association for the Study of the Liver) guidelines. From the beginning of the recruitment, only two dyads were excluded. All the caregivers included acknowledged themselves as the main caregivers (e.g., being the ones who would bring them to the hospital, take care of the appointments, manage the medication regimen).

At the moment of data collection, 19 patients were already hospitalized, and the other 32 had been convened at the hospital to conduct interviews with the practitioner and the psychologist. These two groups were merged because, despite the different circumstances in which the assessment was carried out, any component of the group was met at the same step of the transplant process: the evaluation for inclusion in the waiting list. All patients (included those hospitalized) were deemed sufficiently lucid by the practitioner and the psychologist to compile the questionnaires. Of the total group of patients, 19 suffered from an illness due to a pathology related to alcohol consumption, whereas 32 patients suffered a liver disease due to metabolic or viral cause (e.g., cirrhosis with concurrent hepatitis infection).

The age of patients ranged from 36 to 69 years old and caregivers’ age ranged from 24 to 70 years old. Among the 44 caregivers, 33 were related to the patients as their life partners, three were adult children, six were siblings, and two had other kinds of relationship with the patient. Table 1 summarizes the participants’ socio demographic data.

**Table 1:** Participants’ characteristics.

	Patients	Caregivers
N	51	44
Mean age (SD)	55.7 (8.1)	50.1 (10.1)
Mean years of education (SD)	12.1 (3.7)	11.1 (3.7)
Occupation (%)	80.4	81.8
Kinship (% with the partner)	—	75.0
Pathology (% alcohol)	37.2	—
Hospitalization (%)	37.2	—

Patients and caregivers were informed about the details of the study, and it was explained that the procedure would not affect in any way their admission to the waiting list. The same information was repeated on the printed informed consent form both patients and caregivers were required to sign. Only one dyad refused to participate. Possibly, the rate for patients or caregiver’s refusal has been low because the procedure for compiling the questionnaires did not overlap with the visits with the practitioners. In fact, the procedure required one of the members of the dyad to complete the psychological interview (mandatory as a standard procedure), while the other one would join the collaborator in a different room to compile the questionnaires. Then, they would switch sides and complete the procedure.

To avoid order-effect bias, in half of the dyads, the patient compiled the questionnaires first and in the other half started by doing the interview. The procedure lasted 30–45 min. The ethics committee of Padova Hospital approved the study.

### Data Collection

Socio demographic and clinical data were collected, including current employment situation, kinship in relation to the caregiver, origin of the disease (metabolic or due to alcohol consumption), and the current state of health (hospitalized or not).

Four questionnaires were administered. Both the patient and the caregiver filled in the Symptom Checklist (SCL-90) and Kelly’s Dependency Grids. The Medical Outcomes Study Social Support Survey (MOS-SS) was administered only to patients. The Family Strain Questionnaire-Short Form (FSQ-SF) was compiled only by caregivers.

The SCL-90 (Derogatis, 1983; Sarno et al., 2011) is a self-report inventory with a 5-point *Likert*-*scale ranging from* 0 (*Not at all*) to 4 (*Extremely*). The questionnaire is composed of 10 subscales, which assess psychological symptom patterns of psychiatric and medical patients: somatization (SOM), obsessiveness-compulsiveness (OC), interpersonal sensitivity (IS), depression (DEP), anxiety (ANX), hostility (HOS), phobic anxiety (PHOB), paranoid ideation (PAR), psychoticism (PSY), and sleep disturbances (SLEEP). The SCL-90 also provides a general indicator of the current level of a patient’s psychological distress (Global Severity Index or GSI), an index of the intensity of the symptoms (Positive Symptom Total or PST), and an indicator of the total number of positive symptoms selected by the patient (Positive Symptom Distress Index or PSDI). In the present study, we focused on the subscales of Depression, Anxiety, and the Global Severity Index and Positive Symptom Total indicator.

Kelly’s Dependency Grid, also known as Being Helped Grid (Kelly, 1955; Fransella et al., 2004), is a list of 23 problematic situations (e.g., a time when the participant felt frightened, lonely, or was in poor health). Participants list, in columns, the people who were important to them (the interviewer added “self” as the final resource in the grid) and indicate the person or the people (including him or herself) that they would go for help in each situation. The number of people listed indicates the potential breadth of participants’ social network; the number of resources actually selected, those on whom participants effectively can confide in; the total number of crosses gives a measure of total dependency; the uncertainty column index (UCI) points out the distribution of dependency among different resources; and the dependency percentage participants concentrated on their father and mother, on themselves, and the respective member of their dyad (patient or caregiver) represents a measure of their dependency on each of these resources.

The Italian version of the Medical Outcomes Study Social Support Survey (MOS-SS), (Sherbourne and Stewart, 1991; Giangrasso and Casale, 2014) is a self-administered, multidimensional survey developed for patients with chronic conditions in order to gather information on their perception of social support. This questionnaire has a 5-point Likert scale ranging from 1 (None of the time) to 5 (All of the time), and it investigates four dimensions of the social support provided by the informal caregiver: (1) emotional-informational support (initially intended as separate categories, the former as the expression of empathetic understanding and encouragement to express feelings and the latter as the offering of advice, information, guidance, or feedback), (2) tangible support (the provision of material aid or behavioral assistance), (3) positive social interaction (the availability of other persons to spend some relaxing time together), and (4) affectionate support (involving expressions of love and affection).

The FSQ-SF (Vidotto et al., 2010) consists of 30 dichotomous items (yes/no) that evaluate emotional burden, social involvement problems, the need for knowledge of the disease, the quality of family relationships, and thoughts of death. The overall score is obtained by adding all the positive answers and placing it on a scale of four levels of severity: OK (the caregiver is coping quite well with the situation); Recommended (R) (the caregiver is coping sufficiently well, but there are some indicators of maladjustment, meaning it is worthwhile recommending a psychological consultation in case the “symptoms” get worse); Strongly Recommended (SR) (the caregiver presents an evidence of strain, which certainly needs psychological examination and counseling); and Urgent (U) (the caregiver is greatly strained and at a psychological high risk. It is urgent that s/he is seen by a psychologist or/and by a psychiatrist).

### Data Analysis

The indices of dependency were obtained by analyzing each grid with Bell’s program Gridstat (Bell, 2002). The whole data set was analyzed using R (R Core Team, 2016). A descriptive analysis of the socio-demographic characteristics of participants and of their responses to the questionnaires was performed. Paired *t* tests were used to compare caregivers’ and patients’ mean results. Several associations between the variables were explored through Pearson correlational analyses, both descriptive and inferential.

Regression analyses were conducted in order to test if perceived social support and dependency measures predicted patients’ and caregivers’ symptoms and strain. In order to group the data derived from some indices of patients’ and caregivers’ Dependency Grids, SCL-90, and FSQ-SF, a hierarchical cluster analysis was carried out using the Ward method. This method allowed us to group those data that imply lower deviance increase within the cluster, ensuring the greatest inner cohesion. Whenever analysis required a confrontation within the dyad, the seven patients without a caregiver were not included.

## Results

The rate (*M* = 51.90, SD = 11.00) of patients’ symptoms severity (GSI) was higher [*t*(43) = 2.38, *p* = 0.02] than the caregivers’ rate (*M* = 48.52, SD = 9.42). If we consider the benchmark for moderate to high severity and intensity of symptoms, 31.4 to 43.1% of patients and 20.5 to 27.3% of caregivers, respectively, were over the benchmark. An increase in caregivers’ symptoms was associated with an increase of patients’ symptoms, but the correlation was not significant [*r*(42) = 0.28, *p* = 0.07].

The perceived social support (MOS-SS) reported by patients tended to be generally high, considering the mean score exceeded 4 points over a maximum of 5 (*M* = 4.37, SD = 0.67). An increase in the MOS-SS scale results correlated weakly with a decrease in patients’ symptomatology [*r*(49) = −0.21, *p* = 0.15]. There was no correlation with caregivers’ symptoms [*r*(42) = 0.05, *p* = 0.72].

The FSQ-SF data indicated that caregivers’ strain was at the “Strongly Recommended” level to seek psychological evaluation and support (*M* = 14.45, SD = 6.35). Family strain significantly correlated with caregivers’ [*r*(42) = 0.70, *p* < 0.001] and with patients’ symptoms [*r*(42) = 0.40, *p* = 0.008] but did not significantly correlate with patient’s perceived social support [*r*(42) = 0.07, *p* = 0.63].

Regarding the dependency grids, no differences were found in the extent to which patients and caregivers rely on themselves [*t*(43) = 1.01, *p* = 0.31] or on each other [*t*(43) = 1.30, *p* = 0.20]. Patients’ dependency on the other member of the dyad was significantly higher than their dependency on anyone else [*t*(43) = 4.94, *p* < 0.001], but not higher than the dependency, they had on themselves [*t*(43) = 0.21, *p* = 0.83]. A similar tendency was found in the caregivers [*t*(43) = 2.77, *p* = 0.008; *t*(43) = 0.37, *p* = 0.74].

As shown in Table 2, greater social network breadth (number of indicated resources and selected ones) and diversification of resources (uncertainty index per column) of the patient did not significantly correlate with a decrease in the patient’s symptomatology and only weakly correlated with caregiver’s symptoms and family strain. Patients’ greater concentration of dependency on themselves did not significantly increase their symptoms, caregivers’ symptoms, or family strain. Where the patient showed higher dependency on the other member of the dyad, this did not correlate with the caregiver’s higher symptomatology, whereas caregiver’s symptomatology and family strain did correlate with patient’s higher dependency on someone else. A greater breadth and diversification of the caregivers’ resources did not correspond with a decrease in their own symptoms and strain but did correspond with an increase in patients perceived social support. A higher dependency on self on the part of the caregivers did not relate to a decreased symptomatology or strain. Also, no correlations between caregiver’s dependency on the patient and the general index of symptoms emerged.

**Table 2:** Correlations of dependency measures with patients’ and caregivers’ psychological symptoms (SCL), patients’ perceived social support (MOS), and family strain (FSQ).

		Patients		Caregivers	
		SCL	MOS	SCL	FSQ
Patients	Number of indicated resources	−0.12	−0.08	−0.28	−0.29
	Number of chosen resources	−0.09	0.17	−0.1	−0.11
	Uncertainty by column	−0.04	0.37^*^	0.18	0.11
	Uncertainty by raw	0.17	0.01	−0.12	−0.22
	Dependency on self	−0.02	−0.33^*^	0.04	−0.12
	Dependency on other in the dyad	0.05	0.14	−0.04	0.22
	Dependency on another	0.15	0.17	0.31^*^	0.32^*^
Caregivers	Number of indicated resources	−0.12	0.41^*^	−0.04	0.04
	Number of chosen resources	−0.09	0.34^*^	−0.19	−0.15
	Uncertainty by column	−0.03	0.03	−0.19	−0.25
	Uncertainty by raw	0.13	0.05	0.07	−0.06
	Dependency on self	0.22	−0.13	0.21	0.22
	Dependency on other in the dyad	−0.31^*^	0.15	−0.18	0.20
	Dependency on another	0.19	−0.26	0.14	0.05

* *p < 0.05, levels of significance*.

However, if patients whose illnesses are alcohol- or non-alcohol-related were considered separately, some significant differences emerged. Patients with alcohol-related diseases had a significantly lower number of supportive social resources [*t*(49) = 2.32, *p* = 0.02] and a lower perception of social support [*t*(49) = 2.05, *p* = 0.05]. In this group, family strain negatively correlated with the number of selected resources [*r*(14) = −0.76, *p* < 0.001] and (even if not in a significant way) with the distribution of dependency [*r*(14) = −0.36, *p* = 0.16] on the part of the patients.

With regard to the comparison between MOS-SS measures and dependency grids, perceived social support positively correlated with patients’ uncertainty column index (UCI), and the number of resources indicated and selected by the caregivers (Table 2). Some MOS-SS subscales correlated positively to caregivers’ tendency to rely on the patient. Specifically, tangible support (TAN) and positive support (POS) positively correlated [respectively, *r*(41) = 0.37, *p* = 0.01 and *r*(41) = 0.36, *p* = 0.02] to how much the caregiver relied on the patient. Affectionate support (AFF) positively correlated with the number of resources indicated [*r*(42) = 0.39, *p* = 0.006] and selected [*r*(42) = 0.33, *p* = 0.02] by the caregiver. Also, POS correlated positively with the resources indicated [*r*(42) = 0.34, *p* = 0.02] and chosen by the caregivers [*r*(42) = 0.30, *p* = 0.04].

**Figure 1:**
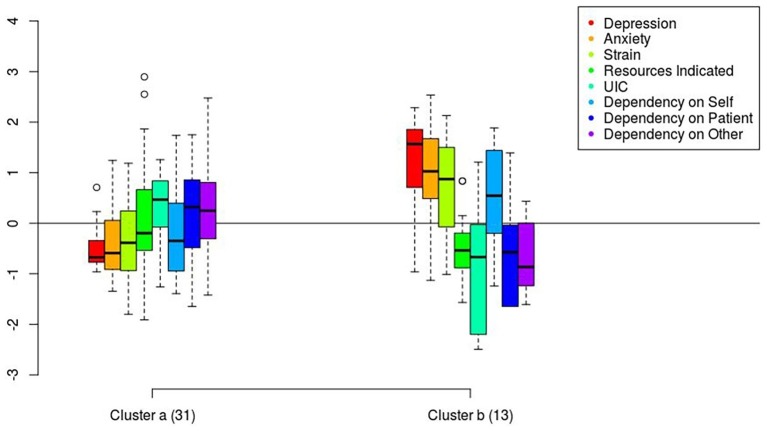
Distribution in the two clusters of caregivers of anxiety, depression, family strain, and dependency measures.

Regression analyses showed that perceived social support and dependency measures did not predict patients’ and caregivers’ symptoms and strain. The cluster analysis showed a distribution in two clusters only for the caregivers. The first cluster, composed of 31 caregivers, showed low levels of depression, anxiety, and strain, together with a high distribution of dependency, low dependency on self, with high levels of dependency on the patient and on another person. The second group, composed of 13 caregivers, showed high levels of depression, anxiety, and strain with a high tendency to rely on themselves more than others (see Figure 1). Neither the variables of age, hospitalization, nor pathology (alcohol related or not) discriminated among the clusters.

## Discussion

Patients and caregivers waiting for a liver transplantation are highly challenged as a dyad, even more so than other organ recipients. The symptoms of encephalopathy that liver failure entails, and the underlying relationship conflicts that may be present if the pathology is linked to substance abuse, might impose a greater burden on the process of waiting (Meltzer and Rodrigue, 2001; Jim et al., 2014; Hansen et al., 2017). The present study is the first to explore the relationship between patient’s and caregiver’s psychological symptoms, perceived social support, and distribution of dependency. Although the group was characterized by some levels of heterogeneity (e.g., the inclusion of patients without a caregiver), the in-depth measures allowed us to portray a picture of the experiences of patients and caregivers during the evaluation for inclusion in transplantation list.

The results are important to pose new questions on how to support efficiently these dyads. Moreover, additional studies could help understanding the differences between this population and other dyads within chronic illnesses. Among the caregivers, women were the majority compared to the males, a not uncommon scenario in this context (Bidwell et al., 2017). Although the sample number did not allow comparisons with male caregivers, literature in the context of organ transplantation suggests women may be more effective in promoting treatment adherence when providing social support to their male partners (Scholz et al., 2012).

Results pointed out that almost a third of patients and a quarter of caregivers showed moderate to high level of psychological symptomatology. This result is in line with previous studies (Cohen et al., 2007; Rodrigue et al., 2011; Malik et al., 2014), which found high psychological distress in caregivers and patients waiting for liver transplantation, but contrary to Malik and colleagues’ (Malik et al., 2014) results, more patients than caregivers were in need of help. A new result of the present study was that an increase in patients’ symptomatology was associated with an increase in caregivers’ symptomatology. So far, a positive association between caregivers’ depression and patient symptoms had been found in cancer (Given et al., 2004), Parkinson (Carter et al., 2008), dementia (Ornstein and Gaugler, 2012), and stroke (Loh et al., 2017) dyads but lacked in dyads facing a chronic, organ-related illness such as heart failure (Bidwell et al., 2017).

Caregivers reported important levels of strain and strongly related to a worsening of their own and patients’ wellbeing. These outcomes are in line with the results of previous studies (Rodrigue et al., 2011; Bidwell et al., 2017) that suggest the importance of supporting caregivers in their daily duties.

In contrast with the results of a previous study (López-Navas et al., 2011), patients reported high levels of perceived social support, but this did not correspond to an increase in their wellbeing nor to a decrease in caregivers’ symptomatology and strain. This result is in line with other two studies (Scholz et al., 2012; Pisanti et al., 2014): the former reported no main effects of provided spousal support on patient intention formation nor adherence behavior; the latter found no association between social support and anxiety. Similarly, a study found that social support was not a mediator between depression and quality of life (Tan et al., 2015). These findings may be understood in the light of previous literature (Goetzinger et al., 2012), which pointed out that patients gave the quality of their relationship higher ratings than their spouses did.

In order to deepen our understanding over the relationship between social support and psychological symptoms in transplantation dyads, the results obtained by the analysis of the dependency grids may be useful. Contrary to what was hypothesized, a greater breadth and a higher distribution of dependency did not correlate with a decrease in patients’ and caregivers’ psychological symptoms and in the family strain. This result is in contrast to the findings of previous studies showing an increase in patients’ wellbeing when they rely on more resources and distribute their dependency (Cipolletta et al., 2012, 2017). However, these studies considered different aspects of patients’ wellbeing and different illnesses. Further research is needed to explore the relationship between illness experience and the distribution of dependency.

The present study offers a contribution in clarifying the relationship between dependency measures and social support in the liver transplantation dyads by showing that patients’ tendency to rely on a broad range of people correlated with higher perceived tangible support. On an interesting note, caregivers who had a broader social network did not feel less stressed or burdened; however, their cared patients perceived a higher social support. Moreover, the broader the caregivers’ social network and the more caregivers relied on the patient, the higher the perceived social support was. Therefore, patients feel more supported when they perceive their caregivers still rely on them. This is a counterintuitive data, which might be understood at the light of these patients’ tendency to rely on themselves. Such a tendency has been found in other clinical populations (Cipolletta et al., 2017, 2019) and deserves to be further explored.

Previous studies (Cipolletta et al., 2013) pointed out that when caregivers depend on others, they can suffer more from psychological symptoms because they are unfamiliar with taking care of others. On the contrary, in the present study, the cluster analysis showed the opposite tendency: when compared with the group of caregivers who relied on themselves, the group of caregivers who tended to rely more on the patients and other resources experienced lower levels of depression, anxiety, and strain. These data suggest that these caregivers, dissimilar to the caregivers in other medical conditions, need to rely more on others than on themselves – a finding which is in line with the previous observation that when caregivers rely on patients, the latter feel more supported. Moreover, the discriminant factor seems to be the distribution of dependency: the group with the higher distribution experiences lower levels of anxiety and depression.

The last finding that deserves to be discussed is the difference between the group of patients with metabolic vs. alcohol-related disease. The second group, as expected, emerged as composed of more vulnerable dyads: to their patients’ narrower social network corresponded an increase in family strain. Patients with an alcohol-related illness also reported a significantly lower perceived social support. In addition to that, the fewer resources their caregivers could actually rely on for help, the more strain they experienced. Although sensibly reduced, the perceived social support appeared to be much more linked to the distribution of dependency in this subgroup. Whenever the patients and the caregivers could rely on a broader and more differentiated network, the patients actually tended to perceive more social support.

These results suggest that these dyads are more similar to those in other clinical conditions (Cipolletta et al., 2012, 2017), but we do not know if this might be explained by a minor interest in social desirability, a major similarity in the kind of interpersonal relationships, or other reasons. Further studies, possibly also using qualitative methods, might explore these aspects.

Alcohol misuse often lead to other social problems such as road traffic accidents (Vu et al., 2019b), drug addiction (Tran et al., 2018), and other risk behaviors (including violence and risky sexual behaviors; Tran et al., 2019a). Psychological interventions including cognitive behavioral therapy, skills training, or motivational interviewing can improve psychosocial well-being of patients with an alcohol-related illness (Tran et al., 2019b). Such interventions can be delivered *via* smartphone applications (Zhang et al., 2016).

The main limitation in this study is the small size of the sample, although this sample size is comparable to previous studies in this area (Goetzmann et al., 2012; Malik et al., 2014). Another limitation is the possibility of social desirability linked to the condition of people inserted in a liver transplantation list. This behavior was noticed in previous studies (Carnrike, 1997; Goetzinger et al., 2012) and may reflect an effort on the patients’ part to minimize the risk of jeopardizing their listing status. Finally, the design was cross-sectional and did not allow for inferences on the direction of the association between variables. Longitudinal follow-up is needed to determine whether and how the association between social support and psychological distress may change over time (i.e., during the pre- and post-transplant periods).

In conclusion, the results of this study may represent an initial contribution to the exploration of the liver transplantation dyads and their peculiar characteristics, even more so as there is a strong possibility that dyads where alcohol abuse is involved will present important differences when compared with those facing a liver failure of different origin. On a more general level, this contribution opens the way for considerations about which similarities and which differences this population shares with patients and caregivers facing other illnesses. From our first observation, patient and caregiver in this dyad do not seem to behave the same way as dyads in other medical conditions. This could be due to a sum of factors typical of the liver transplantation process: uncertainty, fear of being rejected from the listing status (Goetzinger et al., 2012), and tendency to manipulation in patients with alcohol problems (Carnrike, 1997; Mangueira and Lopes, 2016). Nonetheless, these results support the presence of a transactional effect among patient-caregiver dyads (Bidwell et al., 2017) and with it the importance to evaluate the dyad through different and interconnected tools. A deeper knowledge of dyads in organ transplantation will help practitioners in assessing the dyads’ ability to function adequately and identifying situations at risk.

## Data Availability Statement

Publicly available datasets were analyzed in this study. This data can be found here: https://osf.io/cwbjt/.

## Author Contributions

SC gave the main contribution to the theoretical formulation and design of the study. LE, AF, GG, UC, and BV contributed to the concept and design. LE also recruited participants and collected the data. MN contributed to the data analysis. All authors gave the final approval of the version to be submitted.

### Conflict of Interest

The authors declare that the research was conducted in the absence of any commercial or financial relationships that could be construed as a potential conflict of interest.
